# Complexation of Walnut Protein with Adenosine Nucleotides: Effects on Protein Functionality and Novel Insight into the Absorption Mechanism of cAMP

**DOI:** 10.3390/foods15081429

**Published:** 2026-04-20

**Authors:** Lei Zhang, Shanxing Gao, Ye Wang, Jingming Li, Jiachen Zang

**Affiliations:** 1 College of Food Science and Nutritional Engineering, China Agricultural University, Beijing 100083, China; zlei_xj@sina.com (L.Z.); nj_001_48@163.com (S.G.); 2 Network and Information Technology Center, Xinjiang Agricultural University, Urumqi 830052, China; wy@xjau.edu.cn; 3 Sichuan Advanced Agricultural & Industrial Institute, China Agricultural University, Beijing 100083, China; lijingming@cau.edu.cn

**Keywords:** walnut protein, adenosine nucleotides, cyclic adenosine monophosphate, bioavailability, solubility, nutrient delivery, molecular docking

## Abstract

Adenosine nucleotides are vital bioactive molecules with potential applications in functional foods and clinical nutrition; however, their poor membrane permeability limits their bioavailability. The utilization of plant proteins is often hindered by their poor solubility and digestibility. To address these challenges, we developed a strategy involving the formation of complexes between the walnut protein (WP) and four adenosine nucleotides. Spectroscopy, mass spectrometry, cell model, molecular docking, and other experimental techniques were conducted in this study; these methods demonstrated that such a complexation significantly enhanced the solubility of the WP to 3~4 mg/mL, while also enhancing its digestive stability in the gastrointestinal tract by 2~3-fold. Most notably, while all adenosines interacted with the protein matrix, cAMP exhibited a superior absorption efficiency, around 100-fold compared with its linear counterparts. Mass spectrometry and molecular docking were combined to reveal a new absorption mechanism for cAMP with the WP hydrolysate. These findings suggest that the complexation of WP and adenosine nucleotides offers a platform to overcome plant protein limitations and achieve efficient intracellular adenosine delivery, thereby establishing a foundation for its use in the development of functional foods.

## 1. Introduction

Adenosine and its nucleotides, including adenosine monophosphate (AMP), adenosine diphosphate (ADP), and adenosine triphosphate (ATP), are naturally occurring bioactive molecules that play pivotal roles in energy metabolism, immune regulation, and the maintenance of cellular homeostasis. Given these critical physiological functions, adenosine compounds offer significant potential for functional foods and specialized medical nutrition. In sports nutrition, ATP is often termed the “energy currency” and has been investigated for enhancing athletic performance by augmenting muscle ATP reserves, improving microcirculation, promoting protein synthesis, and alleviating fatigue [1]. In medical nutrition for critically ill patients, adenosine aids in modulating immune responses; extracellular adenosine regulates immune cell activity via receptor binding to help control excessive inflammatory reactions [2,3,4]. Additionally, AMP-activated protein kinase (AMPK) activators, including AMP analogs, have been studied for their efficacy in ameliorating insulin resistance and metabolic syndromes [5,6]. Cyclic adenosine monophosphate (cAMP) has drawn particular interest as a key second messenger that regulates intracellular communication. Evidence indicates that exogenous cAMP exerts diverse effects, including enhancing cardiovascular function, boosting immunity, promoting nerve regeneration, and increasing the hematopoietic capacity [7,8]. Beyond physiological benefits, adenosine nucleotides contribute to food flavor; AMP serves as a flavor enhancer due to its synergy with sodium glutamate. Despite these attributes, basic research on the application of adenylates as functional food ingredients remains relatively scarce [9].

The structural features of adenosine nucleotides determine their distinct physicochemical properties. These molecules, composed of adenine, D-ribose, and phosphate groups, share a common motif where adenine is linked to ribose via a β-glycosidic bond (Figure S1). Under a physiological pH, the dissociation of phosphate groups renders the molecules strongly hydrophilic and polyanionic [10,11]. The adenine ring offers aromatic and π-π stacking sites essential for receptor and enzyme recognition, while the phosphate groups facilitate metal chelation and electrostatic interactions with proteins [10]. AMP, ADP, and ATP are linear nucleotides that can be differentiated by their phosphate number, which dictates their charge, size, and energy state. Conversely, cAMP features a cyclic conformation formed by a phosphodiester bond between the 3′ and 5′ hydroxyl groups of ribose, endowing it with specific biological properties and chemical stability [12,13,14]. Despite the potential of these molecules, their poor membrane permeability severely restricts their absorption and bioavailability [15]. Although strategies for permeable analogs have been explored, reports on efficient intracellular delivery systems for natural adenosines remain limited [16,17].

In addition to the challenges with adenosine delivery, the demand for sustainable protein sources is escalating, driven by increasing nutritional and health requirements. Plant proteins have emerged as viable alternatives to animal proteins; however, they often suffer from limitations such as poor solubility and digestibility. Plant proteins have the potential to act as carriers for functional molecules. For example, the encapsulation of curcumin in SPI nanoparticles can effectively protect curcumin against damage from light, oxygen, and high temperatures [18]. Owing to their dynamic reversibility, these nanoparticles can respond to stimuli such as the pH and enzymes in a simulated gastrointestinal digestion environment, achieving a controlled release of active ingredients [19]. Among plant proteins, the walnut protein is distinguished by its well-balanced amino acid profile and high digestibility. It offers significant nutritional value and potential bioactivities; however, it has not been reported to be an adenosine carrier. Based on the structural features of adenosine, we hypothesized that the negatively charged phosphate groups might form robust binding interactions with specific side chains within walnut protein matrices [12,20]. Consequently, the construction of adenosine–plant protein complexes could serve a dual purpose: enhancing the solubility and functionality of the plant protein while simultaneously improving the delivery and absorption of adenosine molecules. To test this hypothesis, we investigated the complexation of the WP with four adenosines—ATP, ADP, AMP, and cAMP—using spectroscopy, mass spectrometry, cell models, molecular docking, and other experimental techniques.

## 2. Materials and Methods

### 2.1. Materials

Defatted walnut meal powder was procured from two suppliers, Guanghua Modern Agriculture Co., Ltd. (Kashgar, China), and Congda Grain and Oil Technology Co., Ltd. (Kashgar, China). All of the adenosine was purchased from Shanghai Macklin Biochemical Co., Ltd. (Shanghai, China). 2,2′-Azino-bis(3-ethylbenzothiazoline-6-sulfonic acid) (ABTS) assay kits were obtained from Solarbio (Beijing, China). Simulated gastric fluid (SGF, USP, pH = 2.0; the concentration of pepsin was 3.2 g/L) and artificial intestinal juice (AIJ, USP, pH = 6.8; the concentration of trypsin was 0.464 g/L) were purchased from Shanghai Yuanye Bio-Technology Co., Ltd. (Shanghai, China). Pepsin and trypsin were purchased from J&K Chemical (Beijing, China). The formic acid (HPLC-grade) and acetonitrile used for the high-performance liquid chromatography (HPLC) analysis were purchased from Sigma (Beijing, China). All other chemicals used were of analytical grade, and all solutions were prepared using ultrapure water.

### 2.2. Preparation of Walnut Protein

The walnut protein was prepared using the alkali extraction and acid precipitation method described by Mao and Hua [21], with slight modifications. Briefly, the defatted walnut powder was mixed with deionized water (1:15, *w*/*v*) under continuous stirring for 2 h. The pH of the dispersion was adjusted to 9.0 using 1.0 mol/L NaOH, followed by heating in a water bath at 45 °C for 2 h. The mixture was centrifuged at 10,000× *g* for 10 min at 4 °C, and the resulting supernatant was collected. Its pH was subsequently lowered to 4.5 by adding 1.0 mol/L HCl under continuous stirring. The mixture was stirred at room temperature for 2 h and centrifuged again at 10,000× *g* for 10 min at 4 °C to collect the precipitate. The protein precipitate was redissolved in deionized water (1:15, *w*/*v*), and the pH was adjusted to 7.0 using 1.0 mol/L NaOH. The solution was dialyzed against deionized water for 36 h (MWCO: 3500 Da), with the water being replaced every 12 h. Finally, stripped walnut protein, devoid of coat polyphenols, was obtained by freeze-drying.

### 2.3. Preparation of Adenosine–Walnut Protein (AWP)

The AWP complexes were prepared using the pH-shifting method according to Jiang et al. [22], with small adjustments. The WP (5 mg/mL) was mixed with small molecules (AMP, ADP, ATP, and cAMP) in deionized water at a protein-to-ligand mass ratio of 5:1. The pH of the mixture was adjusted to alkaline conditions (pH of 10.0, 11.0, or 12.0) using 1.0 mol/L NaOH and stirred for 2 h at room temperature to induce protein unfolding. Subsequently, the solution was gradually neutralized to a pH of 7.0 using 0.1 mol/L HCl to facilitate refolding and ligand encapsulation. Insoluble components were removed by centrifugation at 10,000× *g* for 10 min at 4 °C. The supernatant was then subjected to dialysis (MWCO: 3500 Da) in deionized water at 4 °C for 12 h to remove free salts and unbound small molecules, during which the water was renewed every 4 h. The final reaction stock solution was stored at −20 °C for further analyses.

### 2.4. Solubility Detection of WP and AWP

The protein concentrations were quantified using the bicinchoninic acid (BCA) assay according to the manufacturer’s instructions (Solarbio, Beijing, China). Briefly, 10 μL of the sample or BSA standard (0–2000 μg/mL) was added to a 96-well plate, followed by the addition of 200 μL of BCA working reagent. Following a 30 min incubation at 37 °C under light-protected conditions, the samples were equilibrated to the ambient temperature and the absorbance at 562 nm was recorded using a NanoDrop spectrophotometer. The protein concentrations were quantified by interpolation against a calibration curve prepared with bovine serum albumin (BSA).

### 2.5. SDS Page

A 20 μL protein sample was mixed with an equal volume of loading buffer, and the denatured electrophoresis sample was boiled for 5 min. The product was mixed evenly and then electrophoresed in the electrophoresis tank of a bio rad company. When the indicator reached the upper edge of the separating gel, the voltage was adjusted from 90 V to 120 V. The gel was then separated and stained with Coomassie brilliant blue (R-250).

### 2.6. ABTS Clearance Rate of WP and AWP

To prepare the ABTS^+^· working reagent, ABTS was dissolved in 25 mM Tris-HCl buffer (pH of 7.0) to obtain a 7 mM solution, which was then reacted with an equal volume of 2.45 mM potassium persulfate. The mixture was subsequently stored in the dark at 4 °C for 12–16 h to allow for radical formation. The resulting solution was then adjusted with Tris buffer to an absorbance of 0.70 ± 0.02 at 734 nm. For an activity assessment, 3 μL of the sample was introduced into 237 μL of the working reagent and allowed to react under ambient light at room temperature for 30 min. The absorbance was measured at 734 nm using a Cary 50 Bio UV–Vis spectrophotometer (Varian, Palo Alto, CA, USA) (*AE*). The blank control consisted of 3 μL of Tris buffer substituted for the test sample, combined with 237 μL of the diluted ABTS solution (*AB*). The percentage of ABTS^+^· inhibition was calculated using the following formula [23].

(1) The rate of inhibition=[(AB−AE)/ AB]×100

where *AB* is the absorbance of the blank sample and *AE* is the absorbance of the mixture.

### 2.7. Determination of Surface Hydrophobicity

The surface hydrophobicity was determined using 8-anilino-1-naphthalenesulfonic acid (ANS) as a fluorescent probe. Protein samples were prepared through a stepwise dilution in 0.01 M phosphate buffer (pH of 7.0), establishing a concentration series spanning 0.05–0.5 mg/mL. Aliquots of 4 mL of each dilution were supplemented with 20 μL of 8 mM ANS solution. The fluorescence intensity (FI) was recorded on a fluorescence spectrometer with the excitation and emission wavelengths set to 390 nm and 470 nm. The surface hydrophobicity index (H0) was derived from the slope of the initial linear segment in the plot of FI against the protein concentration [24].

### 2.8. Ultraviolet (UV) Full-Wavelength Scanning and Circular Dichroism (CD) Spectra

UV–Vis absorption spectra of the WP and AWP solutions were acquired using a Varian Cary 50 spectrophotometer (Varian, USA) to characterize the protein and nucleic acid components [25]. Briefly, WP and AWP (10.0 mg each) were dissolved in 10.0 mL ultrapure water. The UV–Vis spectra of the WP and AWP were measured from 260 to 700 nm at room temperature, with water as the reference [26]. Far-UV CD spectra were recorded from 190 to 240 nm at a protein concentration of 0.1 mg/mL in pure water, with the band width set to 1 nm and the path length set to 0.1 cm.

### 2.9. Fluorescence Spectra

Intrinsic fluorescence spectroscopy measurements were conducted according to the methodology outlined by Sun et al. [27] with certain modifications. The protein concentration used for the measurement was 0.5 mg/mL. Fluorescence spectra were recorded for λexc = 280 nm and λemi = 300–400 nm.

### 2.10. In Vitro Simulated Gastrointestinal Digestion

The samples were subjected to an in vitro gastrointestinal digestion model following the protocol of Brodkorb et al. [28]. According to the manufacturer’s instructions, SGF is primarily composed of diluted hydrochloric acid and pepsin, with a pH = 2.0; SIF is formulated using a phosphate buffer and trypsin, with a pH = 6.8. Gastric digestion was performed as follows: (1) The sample solution was mixed with SGF at a 1:1 (*v*/*v*) ratio and supplemented with pepsin at a concentration of 2000 U/mL. Gastric digestion was simulated at a pH of 3.0, 37 °C, and 200 rpm for 120 min. The reaction was terminated by adding 10 μL of 5 M NaOH, and a portion of the sample was collected into a vial. (2) The gastric chyme was then mixed with SIF at a 1:1 (*v*/*v*) ratio and supplemented with trypsin at 100 U/mL. Intestinal digestion was simulated at a pH of 7.0, 37 °C, and 200 rpm for 120 min. The sample tube was immersed in a boiling water bath for 5 min to inactivate the enzymatic reaction. (3) Subsequently, the digest was stored at −20 °C for further analysis. It should be pointed out that enzymes except for protease, like gastric lipase and amylase, were omitted to isolate proteolysis and minimize matrix interference in the defatted system. It would be more accurate to compare the difference between natural and complexed WP. While this deviation from INFOGEST 2.0 enhances analytical precision for protein-focused assays, it is a limitation as it lacks the synergistic effects of a complete physiological digestive environment.

### 2.11. Transepithelial Transport of WP and AWP Solutions by Caco-2 Cells

Caco-2 cells were obtained from Zhejiang Meisen Cell Technology Co., Ltd. (Jinhua, China). The cells were cultured in 5 mL of Dulbecco’s modified Eagle medium (DMEM; No. C11095500BT, GIBCO, Waltham, MA, USA) supplemented with 20% fetal bovine serum (FBS; No. A5256701, GIBCO) and 1% penicillin–streptomycin liquid (No. P1400, Solarbio). The cultures were maintained at 37 °C in a humidified atmosphere of 5% CO_2_ and 95% air. The culture medium was refreshed daily, and cells were passaged upon reaching 80–90% confluence.

Caco-2 cells in the logarithmic growth phase were digested with 1 mL of a 0.25% trypsin–EDTA solution (No. T1300, Solarbio) at 37 °C for approximately 2–3 min, and digestion was terminated by adding 2 mL of complete DMEM. The cell suspension was transferred to a sterile centrifuge tube and centrifuged at 200× *g* for 5 min at room temperature. After discarding the supernatant, the pellet was resuspended in DMEM, and the cell concentration was determined using a hemocytometer. The suspension was then adjusted to a fine density of 1 × 10^5^ cells/mL.

This suspension was seeded onto the apical side of 12-well Transwell plates (PC, 0.4 μm Beyotime), and 1.5 mL of DMEM was added to the basolateral compartment. The plates were incubated at 37 °C in a 5% CO_2_ atmosphere. The culture medium in both compartments was replaced every two days for 10–21 days. The transepithelial electrical resistance (TEER) was measured using a Millicell ERS-2 system (Millipore, Bedford, MA, USA). Monolayers with TEER values ≥ 300 Ω·cm^2^ were used for the transport experiments.

Caco-2 cells were seeded in Transwell chambers following a previously described protocol [29]. Prior to the transport assays, the Caco-2 monolayers were rinsed three times with Hank’s balanced salt solution (HBSS, pH of 7.4). The Caco-2 transport experiments were conducted using the fraction obtained from the in vitro simulated gastrointestinal digestion of the AWP complexes, which was subsequently filter-sterilized (0.22 μm). For the transport assays, 0.5 mL of this digested fraction (1 mg/mL in HBSS) was added to the apical compartment, and 1.5 mL of HBSS was added to the basolateral compartment of the Transwell inserts. At 30, 60, and 120 min during incubation, 0.6 mL aliquots were withdrawn from the basolateral compartment, transferred to Eppendorf tubes, and immediately replaced with an equal volume of fresh HBSS.

### 2.12. Determination of Adenosine Using High-Performance Liquid Chromatography (HPLC)

The HPLC (LC-40, Shimadzu, Kyoto, Japan) equipped with SPD-M20A detectors was employed for the adenosine analysis; chromatographic separation was performed on a C18 column (250 mm × 4.6 mm, 5 μm; Thermo Fisher Scientific, Waltham, MA, USA) at 30 °C with a flow rate of 0.5 mL/min and a 10 μL injection volume. The mobile phase comprised 15 mmol/L KH_2_PO_4_ (solvent A) and 10% (*v*/*v*) methanol in ultrapure water (solvent B), with the following gradient: 0–17 min, 10% B; 17–18 min, 50% B; 18–19 min, 80% B; 19–21 min, 80% B; 21–23 min, 90% B; and 23–25 min, 10% B. Detection was at 254 nm. Adenosine was identified by matching its retention time to that of an authentic reference standard and quantified using the external standard method based on the peak area.

### 2.13. Peptide Identification and Protein Sequencing Using Liquid Chromatography–Tandem Mass Spectrometry (LC-MS/MS)

The detection method was modified based on the method described by Wang et al. [30]—LC-MS/MS (Vanquish Neo/Orbitrap Exploris 480, Thermo Fisher Scientific, Waltham, MA, USA). The peptides were enriched on PepMap Neo C18 (300 μm × 5 mm, 5 μm, Thermo Fisher Scientific) and separated on an in-house-packed analytical column, Reprosil-Pur 120 C18-AQ (100 μm i.d. × 170 mm, 1.9 μm; Dr. Maisch, Ammerbuch, Germany). The mobile phase comprised 0.1% formic acid (FA) (solvent A) and 0.1% FA with 80% acetonitrile (ACN) (solvent B). Gradient elution was programmed as follows: 0–1.8 min, 6% B; 1.8–2 min, 6% B; 2–17 min, 6.5% B; 17–24.5 min, 23% B; 24.5–25 min, 36% B; and 25–30 min, 99% B. The injection volume was 1 μL and the flow rate was 400 nL/min.

MS data were acquired in DDA mode. The MS scan range (*m*/*z* 300–1800) was acquired at a 60,000 resolution, an AGC target of 300%, and a maximum IT of 50 ms. The MS/MS spectra were obtained at a 15,000 resolution, an AGC target of 100%, and a 1.5 s cycle time. The raw mass spectrometry files were searched against the previously constructed target protein database using PEAKS Studio 13.1 (Bioinformatics Solutions Inc., Waterloo, ON, Canada). The following parameters were applied: trypsin/P and Lys-C digestion (cleavage after K/R, ≤2 missed cleavages); a fixed modification of carbamidomethyl (C); variable modifications of oxidation (M) and acetyl (protein N-term); precursor and fragment mass tolerances of ±10 ppm and ±0.02 Da, respectively; and an FDR cutoff of 1% at both the PSM and protein levels.

### 2.14. Molecular Docking

The protein structure (PDB ID: 7QCL) was downloaded from the PDB library, which can be open in Discovery Studio 2017R2. After removing irrelevant small-molecule ligands and ions, the Define Site from Receptor Cavities function was used to obtain docking sites X, Y, and Z, which were 229.785088, 265.737016, and 196.503667, respectively. Molecular docking and configuration scoring were performed using HPEPDOCK 2.0 [31] based on the scoring function ITScorePP.

### 2.15. Statistical Analysis

The results are expressed as the mean ± SD from three independent biological replicates. The normality of the data was verified by the Shapiro–Wilk test (*p* > 0.05). The data underwent an ANOVA followed by the Student–Newman–Keuls test for multiple comparisons. Statistical significance was set at *p* < 0.05. All statistical analyses were conducted using SPSS 26.0 (IBM Corp., Armonk, NY, USA).

## 3. Results and Discussion

### 3.1. Solubility Analysis of AWP

**Figure 1:**
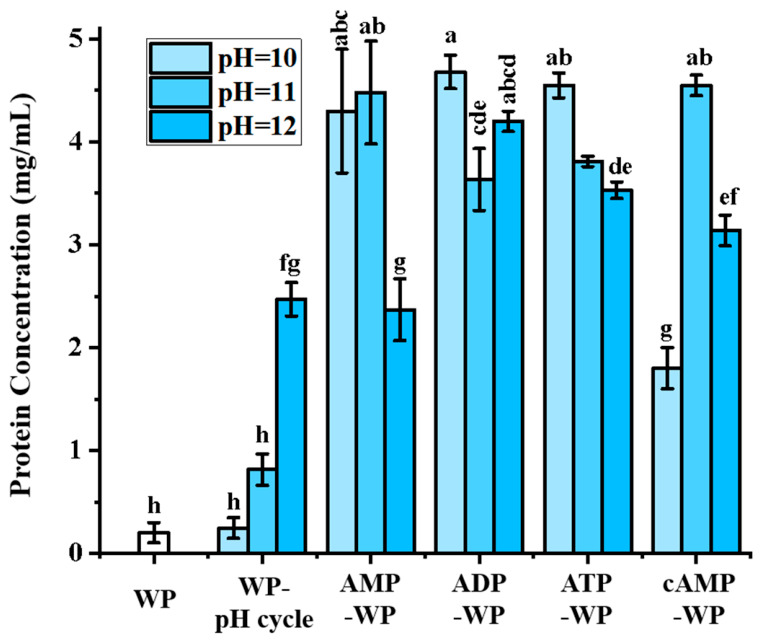
Protein concentrations of untreated WP (Walnut Protein, WP), pH-treated WP (WP-pH cycle), AMP-treated WP (AMP-WP), ADP-treated WP (ADP-WP), ATP-treated WP (ATP-WP), and cAMP-treated WP (cAMP-WP). Different lowercase letters indicate statistical significance (*p* < 0.05).

An alkaline treatment is a well-established method of improving plant protein solubility by modulating the surface charge density and electrostatic repulsion [32]. As depicted in Figure 1, the WP samples subjected to an alkaline treatment at a pH of 10, 11, and 12 exhibited a significantly improved solubility compared to the native control sample. The observed enhancement was pH-dependent, with the solubility increasing at higher pH values, and it is attributed to the extreme pH conditions inducing more severe protein denaturation and unfolding, which exposes buried hydrophilic amino acid residues and increases the surface hydrophilicity. These results align with reports on soy and pea proteins, in which alkaline environments facilitated aggregate dissociation and promoted side chain ionization [33,34].

To investigate the synergistic effects of nucleotide additives, the WP samples were treated under identical alkaline conditions with various adenosine molecules, including ATP, ADP, AMP, and cAMP. Following treatment, the samples underwent a pH-shifting cycle to return to a neutral medium. Incorporating adenosine derivatives enhanced the solubility relative to the alkaline treatment alone. The solubility of the AWP samples peaked at 4.6 mg/mL, representing a 20-fold increase over the untreated WP. However, the solubility trends showed complex dependencies on both the specific adenosine derivative and the treatment pH. Specifically, AMP-WP and cAMP-WP exhibited an optimal solubility at a pH of 11, whereas ADP-WP and ATP-WP demonstrated a superior solubility at a pH of 10. This divergence may be linked to the structural characteristics of the phosphate groups within adenosine molecules. Adenosine phosphates are highly hydrophilic because of their negative charges; thus, their introduction enhances protein solubility. Moreover, it has been well documented that the solubility of free adenosine phosphates in aqueous solutions follows the order ATP > ADP > AMP. This order is primarily determined by the number of phosphate groups, which impart a greater hydrophilicity and electrostatic repulsion, despite the increased molecular weight [35]. The interaction between these highly soluble phosphate moieties and the unfolded protein matrix likely contributes to the observed enhancement in solubility.

Given that alkaline treatments involve extreme pH levels, it was hypothesized that the improved solubility might be partially driven by the degradation of WP into smaller peptides. However, the SDS-PAGE analysis revealed that the electrophoretic bands of the adenosine-conjugated WP remained virtually identical to those of the untreated and alkaline-only treated samples (Figure S2). This indicates that extensive hydrolysis did not occur during the reaction. Consequently, the significant enhancement in solubility can be predominantly attributed to conformational changes in the protein structure, such as an increased surface hydrophilicity and electrostatic repulsion induced by adenosine molecule binding, rather than changes in the molecular weight.

### 3.2. Structural Characterization of AWP

**Figure 2:**
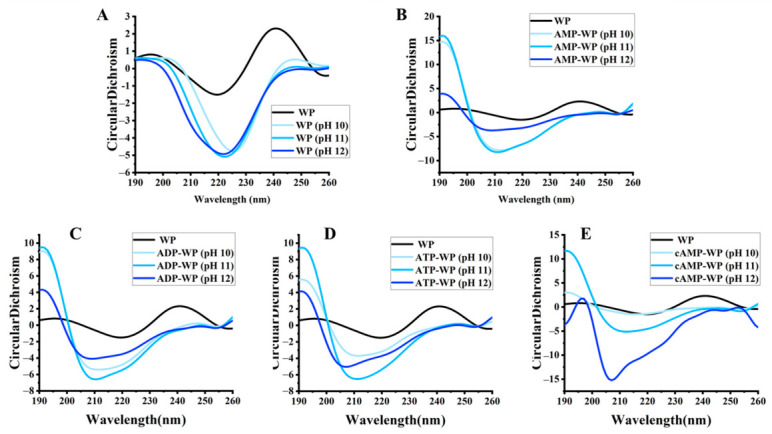
CD spectra of (**A**) untreated walnut protein (WP); (**B**) AMP-treated WP (AMP-WP); (**C**) ADP-treated WP (ADP-WP); (**D**) ATP-treated WP (ATP-WP); and (**E**) cAMP-treated WP (cAMP-WP) at different pH values.

CD spectroscopy provides information on the secondary structural composition of proteins in solution. As shown in Figure 2, the alkaline-treated WP exhibited a marked decrease in ellipticity between 208 and 222 nm, suggesting that the alkaline environment induced the partial unfolding of the native protein. This unfolding was accompanied by a reduction in the ordered secondary structures, specifically α-helices and β-sheets. Interaction with adenosine derivatives had a greater impact on the WP secondary structure than the pH treatment alone. As illustrated in the subsequent spectra, complexation caused a significant decrease in the α-helix and β-sheet content, accompanied by a substantial increase in random coil structures. This uniform trend across the four adenosine types suggests that the interactions between the WP and adenosines destabilize the native secondary structure. These observations align with previous studies, indicating that ligand binding can disrupt the hydrogen bonding that is essential for maintaining α-helical and β-sheet conformations [36,37].

**Figure 3:**
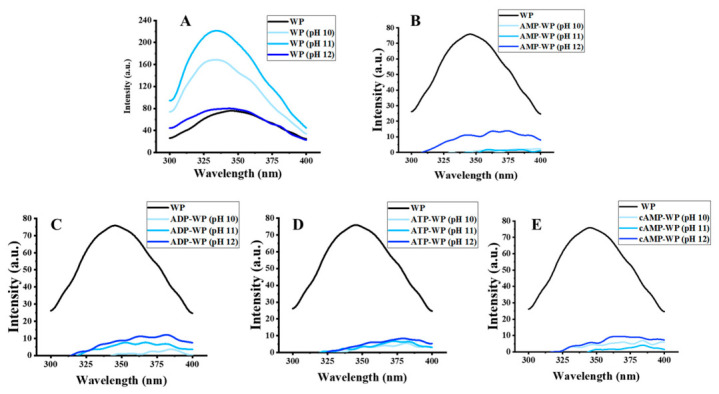
Fluorescence spectra of (**A**) untreated walnut protein (WP); (**B**) AMP-treated WP (AMP-WP); (**C**) ADP-treated WP (ADP-WP); (**D**) ATP-treated WP (ATP-WP); and (**E**) cAMP-treated WP (cAMP-WP) at different pH values.

Following a secondary structure analysis, it is crucial to characterize the changes in the tertiary structure induced by pH-shifting in the presence of adenosine. Intrinsic fluorescence, primarily derived from tryptophan (Trp) residues, is extensively used to probe protein–ligand interactions because of the high sensitivity of Trp to changes in the local microenvironment [38,39]. WP is rich in Trp residues, exhibiting a strong fluorescence emission band at 324 nm upon excitation at 280 nm. With increasing alkaline severity, a progressive decrease in FI was observed, confirming Trp’s sensitivity to alkaline environments (Figure 3). FI did not recover upon returning the pH to neutral, suggesting that the alteration in the tertiary structure was irreversible. Upon the addition of adenosine, the fluorescence was quenched. This phenomenon is distinct from the spectral changes typically observed in pH-shifted samples or complexes formed via non-covalent bonding, where red shifts or partial quenching are more common [23,40]. The complete quenching observed here implies that adenosines may interact with the WP, destroying the native microenvironment surrounding Trp residues.

**Figure 4:**
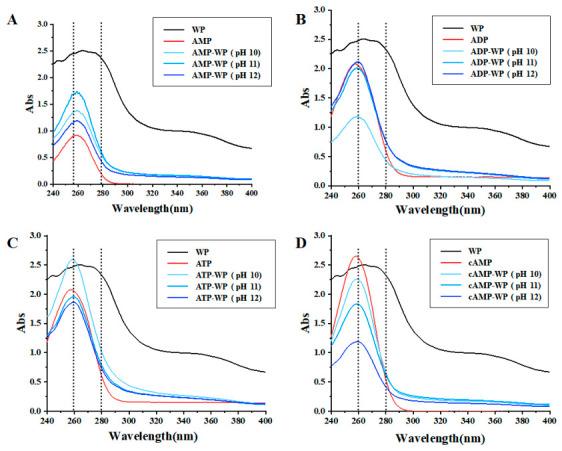
UV–Vis absorption spectra of (**A**) AMP-treated WP (AMP-WP); (**B**) ADP-treated WP (ADP-WP); (**C**) ATP-treated WP (ATP-WP); and (**D**) cAMP-treated WP (cAMP-WP), adenosine and untreated walnut protein (WP) at different pH values.

Proteins characteristically exhibit a UV–Vis absorption peak at 280 nm, attributed to the conjugated π-π electron systems of aromatic amino acids (tryptophan, tyrosine, and phenylalanine). In their native tertiary structure, these residues are often buried within the hydrophobic core; consequently, the peak’s position and intensity are sensitive to the microenvironment polarity. In contrast, adenosine displays a distinct absorbance maximum at 260 nm resulting from a π–π * transition in the adenine purine ring. As illustrated in Figure 4, the adenosine treatment caused the characteristic 280 nm peak of WP to disappear, while the 260 nm peak intensity varied significantly. The loss of the protein peak and changes in the adenosine peak indicate strong interactions that modify the chromophores’ electronic environment.

Structurally, the adenine ring serves as a bulky hydrophobic moiety. Upon attachment to the protein surface, this group likely penetrates the hydrophobic core to minimize aqueous contact. This insertion can severely disrupt the non-covalent forces stabilizing the native conformation, such as hydrogen bonds, hydrophobic interactions, and van der Waals forces. Consequently, this leads to the extensive unfolding of the tertiary structure. As the structure unfolds, buried tryptophan and tyrosine residues become exposed to the polar aqueous environment. This exposure accounts for the CD and fluorescence data, confirming that the tertiary structure of WP was extensively disrupted by complexation with adenosine.

### 3.3. Physical and Chemical Characterization of AWP

**Figure 5:**
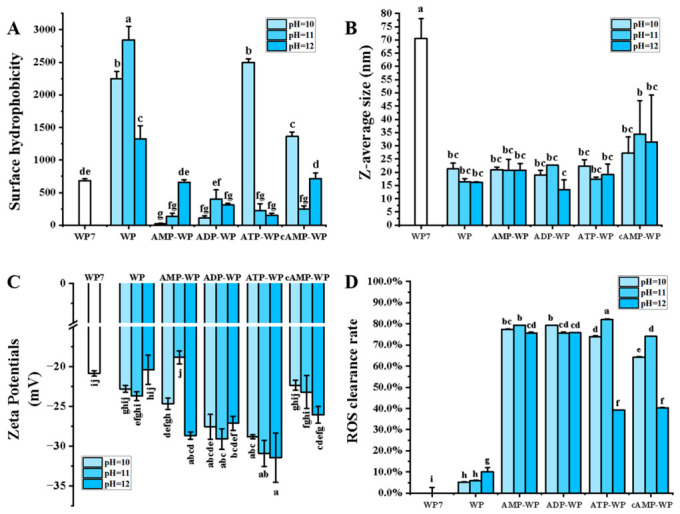
Surface hydrophobicity (**A**), particle size (**B**), surface charge (**C**), and antioxidative capacity (**D**) of WP and AWP at different pH values. pH value is 10 to 12 from light blue to dark blue. WP (untreated walnut protein), ATP-treated WP (ATP-WP), AMP-treated WP (AMP-WP), ADP-treated WP (ADP-WP), and cAMP-treated WP (cAMP-WP), pH 7-treated WP (WP7). Different lowercase letters indicate statistical significance (*p* < 0.05).

The surface hydrophobicity (H0) and surface charge are critical parameters that determine protein functionality, including solubility, emulsification, and digestibility. Figure 5A shows that the adenosine-modified WP exhibited a significantly lower H0 index compared to the unmodified WP (*p* < 0.05), resulting from the hydrophilic nature of adenosine molecules rich in ribose hydroxyl and phosphate groups. Complexation with these polar moieties shields hydrophobic surface patches, enhancing the overall hydrophilicity [41,42]. The untreated WP also displayed a relatively low H0, likely due to the natural tendency of the WP to aggregate in aqueous solutions. In aggregated states, hydrophobic clusters are buried within the protein core or at interfaces, making them inaccessible to the hydrophobic fluorescence probe. ATP-WP and cAMP-WP at a pH of 10 showed higher H0 values, which might have been caused by incomplete complexation between the two molecules at such a pH value. The particle size analysis (Figure 5B) supports this, showing that the native WP had a large mean diameter of approximately 70 nm. Conversely, pH-shifting treatments, with or without adenosine, dissociated these aggregates, significantly reducing the average particle size.

In terms of the surface charge, adenosine complexation significantly altered the electrostatic properties of the WP. Complexation targets lysine residues, blocking their positively charged amino groups and thereby increasing the protein’s net negative charge. Additionally, incorporating negatively charged phosphate groups (in ATP/ADP) and hydroxyl groups contributes to this shift [43]. Figure 5C shows that the adenosine-complexed samples displayed significantly lower zeta potentials (more negative values). The ATP-WP conjugate exhibited the lowest zeta potential, approximately −32 mV. This higher negative charge density stems from the triphosphate group of ATP, which provides more ionizable groups than AMP or cAMP. The heightened surface charge increased the electrostatic repulsion between protein molecules, aligning with the improved solubility noted earlier.

Adenosines and derivatives are widely recognized for their bioactive properties, particularly their antioxidative capacity, which arises from their ability to scavenge free radicals and chelate pro-oxidant metal ions [44]. Nevertheless, whether complexation preserves these bioactive attributes remains uncertain, especially under harsh alkaline conditions. To address this, the antioxidant activities of adenosine–WP complexes were assessed. As depicted in Figure 5D, despite undergoing alkaline treatment during complex formation, the four adenosine-modified WP samples retained substantial antioxidant activity, which corresponds to the ABTS antioxidant assays. Conversely, the native and pH-shifted WP controls exhibited a negligible capacity. Thus, this synergy not only enhances the nutritional value of WP by incorporating a functional ingredient, but also exploits WP’s structural modifications (improved solubility and stability) to potentially facilitate adenosine delivery and bioavailability.

### 3.4. Effect of Adenosine on the Digestibility of AWP

Digestibility is a critical factor in plant protein utilization, and is often limited by a low solubility and irregular aggregation states. The previous results confirmed that adenosine binds to WP, inducing significant structural and physicochemical alterations. To determine whether such structural modulations could improve WP digestibility, we evaluated and compared the digestibility of the native WP and AWP by INFOGEST 2.0.

**Figure 6:**
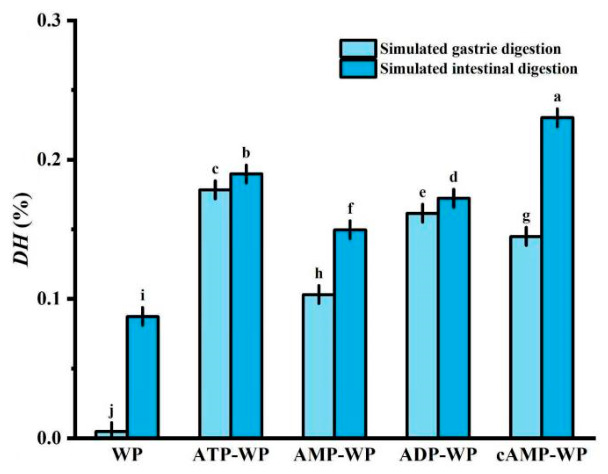
DH of the WP and adenosine-complexed WP (untreated walnut protein, WP) for both the gastric (light blue) and intestinal (dark blue) digestion phases. ATP-treated WP (ATP-WP), AMP-treated WP (AMP-WP), ADP-treated WP (ADP-WP), and cAMP-treated WP (cAMP-WP). Different lowercase letters indicate statistical significance (*p* < 0.05).

Figure 6 illustrates the degree of hydrolysis (DH) for both the gastric (light blue) and intestinal (dark blue) phases. We chose to consider the characteristics of the samples treated with a pH of 11 for this experiment. During gastric digestion, protein hydrolysis is governed by acidic conditions and pepsin activity. This process was relatively inefficient for the untreated WP, likely owing to its compact native structure and low accessibility to pepsin. In the intestinal phase, trypsin plays a predominant role. Trypsin had a more pronounced effect on the untreated WP, increasing the DH to approximately 8.5%. Conversely, the adenosine-conjugated WP samples exhibited a significantly higher susceptibility to hydrolysis. The cAMP-WP conjugate achieved the highest DH, reaching 22.5%.

The observed enhancement in digestibility was attributed to conformational changes induced by adenosine. As previously established, an alkaline treatment and subsequent binding severely disrupt the native tertiary structure of WP. Upon shifting the pH back to neutral, steric hindrance and structural interference imposed by the conjugated adenosine molecules prevent the protein from reassembling into its original dense, tightly packed conformation. Such a loose conformation is inherently more flexible and solvent-exposed. Consequently, enzymatic cleavage sites that were originally buried within the hydrophobic core become more accessible to proteases such as pepsin and trypsin. Furthermore, the introduction of hydrophilic adenosine moieties may improve the protein solubility, facilitating enzyme–substrate interactions, which are often the rate-limiting step in protein digestion [45].

### 3.5. Absorption of Adenosine Through Caco-2 Cell Monolayers

Adenosine and its derivatives possess a wide spectrum of nutritional and pharmacological functions [46]. However, their clinical application is often hindered by their low membrane permeability and poor bioavailability [15]. Recently, macromolecules such as proteins and polysaccharides have garnered attention as natural carriers capable of enhancing the intestinal absorption of bioactive compounds. Consequently, we aimed to investigate whether complexation with WP could improve the absorption profile of exogenous adenosine following gastrointestinal digestion.

The Caco-2 cell monolayer model is a gold-standard in vitro system widely used to evaluate intestinal permeability and drug transport [47]. In this study, the apparent permeability of the four adenosine derivatives was assessed at different time points (Table 1). Minimal amounts of free adenosines (ATP, ADP, AMP, and cAMP) were detected on the basolateral side, confirming their limited passive diffusion across the intestinal epithelium. We determined the content of cAMP and other adenosines by comparing their peak area with the standard molecule. This is a commonly used method for HPLC. However, the transport efficiency of the ATP-WP, ADP-WP, and AMP-WP complexes remained comparable to those of the free molecules. This suggests that it failed to facilitate ATP, ADP, or AMP transport, despite improving the protein carrier digestibility. Notably, the cAMP-WP complex exhibited a distinct trend; cAMP transport was enhanced compared to the other groups, reaching a cumulative concentration of approximately 1.77 mg/mL within the first 30 min. An additional fraction (1.36 mg/mL) was detected in the subsequent 30 min, indicating a sustained, but gradually decreasing, transport rate. These results show that WP might promote the transcellular transport of cAMP across Caco-2 monolayers. Consistent with previous studies on macromolecule–small molecule interactions [48], we hypothesized that cAMP remains associated with WP peptide fragments even after digestion. This allows transport to occur via protein-specific uptake pathways rather than solely through passive diffusion.

The distinct enhancement observed for cAMP, unlike other adenosines, warrants detailed discussion. The absorption of protein–polyphenol or protein–nutrient complexes is influenced by factors such as the surface charge, molecular weight, steric configuration, and hydrophobicity [49]. Although cAMP has a slightly lower molecular weight than ATP, this alone cannot explain the discrepancy. Furthermore, it is hard to confirm the structural changes in adenosines during in vitro digestion and absorption. Thus, future experiments are required to elucidate the detailed mechanism.

### 3.6. Absorption Based on Mass Spectrometry

**Figure 7:**
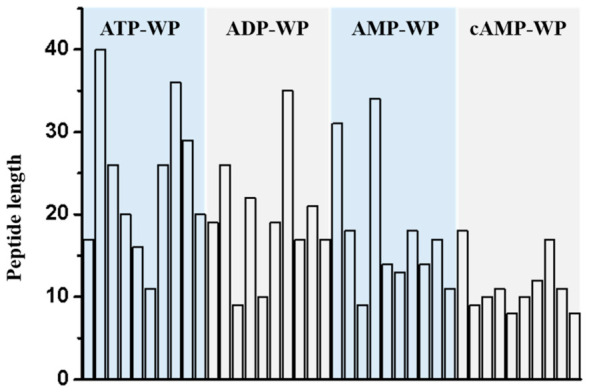
Peptide length of the identified peptides obtained from AWP with high scores.

To speculate on the possible mechanism, a mass spectrometry (MS) analysis was conducted. As shown in Figure 7, the trend indicated that the cAMP-WP complex was digested into peptides of significantly shorter lengths than the other complexes. The average peptide lengths of the digested ATP-WP, ADP-WP, AMP-WP, and cAMP-WP were 24.1, 19.5, 17.9, and 11.4, respectively. Furthermore, four types of interactions between adenosine and the WP were identified: ribosylation, oxidation, carbamidomethylation, and acetylation (Supplementary Table S1). Ribosylation was the predominant interaction in the cAMP-WP complex, likely attributed to the unique cyclic structure of cAMP. Specifically, the cyclic structure might sterically hinder the phosphate group, rendering the ribose moiety the primary reactive site for attachment to the protein. Unlike the linear nucleotides that may form extensive shielding networks, the compact cAMP conformation induces less steric hindrance, thereby exposing more enzymatic cleavage sites and facilitating the deep hydrolysis of the protein into shorter peptides.

We initially hypothesized that the cyclic structure of cAMP allows it to partially neutralize the WP’s surface charge via its amino group [50]. This charge masking could theoretically reduce the electrostatic repulsion between the complex and the negatively charged cell membrane, promoting cellular uptake. We also postulated that cAMP binding modulates WP surface hydrophobicity, as a moderate increase often enhances peptide permeability through the gastrointestinal mucous layer, facilitating epithelial access. To test this, we analyzed the predicted isoelectric point (pI) and average hydropathicity of peptides from digested adenosine–WP complexes (Figure S3). However, no significant correlations or regular distribution patterns were observed for electrostatic or hydrophobic properties.

As the surface charge and hydrophobicity did not fully account for the results, we turned to the mucus barrier—a critical selective interface for nutrient absorption. Mucus forms a protective viscoelastic gel composed of large glycoproteins (mucins) on wet epithelial surfaces, including the GI tract [51]. This layer critically influences bioavailability, as mucins can adhere to drugs and modulate transport [52]. Thus, we analyzed peptide–mucin interactions via molecular docking. Four representative peptides were selected from the MS-identified pool, showing a length gradient, but sharing similar electrostatic and hydrophobic properties. The docking results revealed that the shortest peptide bound to the mucin protein (PDB 7QCL) [53] with a score of approximately −104. Conversely, the other three peptides (~10, 20, and 30 residues) demonstrated significantly stronger binding, scoring around −200. These findings imply that longer peptides possess a higher mucin affinity, which may hinder their passage through the mucus network.

**Figure 8:**
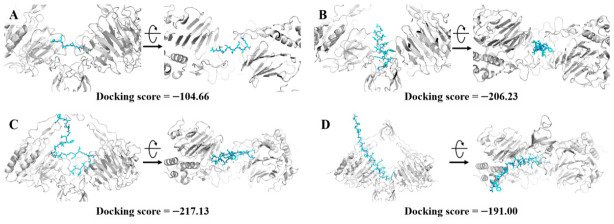
Molecular docking results of four selected peptides with mucin protein: (**A**) peptide length = 10; (**B**) peptide length = 20; (**C**) peptide length = 30; (**D**) peptide length = 40.

Beyond binding energy, steric hindrance critically influences absorption. As shown in Figure 8, longer peptides cross-link with mucin in a spatial interaction that effectively traps them within the mucus gel. This finding contrasts with our previous study on polysaccharides, where a polysaccharide–cAMP complex improved absorption via mucin-adhesive properties. Conversely, in this protein-based system, peptide carriers may appear to enhance absorption by facilitating a more flexible route through mucus, primarily due to their shorter chain lengths.

The above results reveal two distinct differences. The release mechanism from adhered macromolecules—feasible for polysaccharides—may not apply to protein carriers that form complexed adducts. Second, polysaccharides and protein hydrolysates differ significantly in their molecular weight and size. Different molecules penetrate the mucous layer with their own strategies, respectively. In our research, smaller-sized-peptides with a more flexible penetrating ability might be an explanation for the higher bioavailability of cAMP. However, more experiments are needed to elucidate a universally recognized mechanism and additional details on the absorption pathway. Such experiments could include peptide synthesis, a structural analysis of protein–adenosine complexes, and in vivo animal experiments.

## 4. Conclusions

Adenosine nucleotides are vital bioactive molecules with significant potential for functional foods and clinical nutrition. However, their therapeutic efficacy is often limited by their poor membrane permeability and low bioavailability, while the utility of plant proteins such as the WP is restricted by low solubility and digestibility. This study presents a novel strategy to address these dual challenges by constructing complexes between the WP and four adenosine nucleotides.

Complexation significantly improved the solubility and antioxidant capacity of the WP, while enhancing its digestive stability within the gastrointestinal tract. The nucleotides exhibited a notable difference in their absorption efficiency: cAMP displayed a superior bioavailability compared to its linear counterparts (ATP, ADP, and AMP). This enhanced uptake might have resulted from cAMP’s unique cyclic structure, which favors specific interactions, predominantly ribosylation, with the protein matrix. Consequently, upon digestion, the cAMP-WP complex might yield peptides of significantly shorter average lengths than other adenosine complexes. To isolate proteolysis and minimize matrix interference for LC-MS/MS analysis, gastric lipase and amylases were omitted from the INFOGEST 2.0 protocol. While this modification ensures analytical precision for the defatted walnut protein-nucleotide complexes, it is a methodological limitation that may not fully replicate complete physiological enzymatic interactions.

In conclusion, the WP–adenosine complexation strategy serves as a multifunctional platform that effectively mitigates the limitations of walnut proteins. By establishing a structure-dependent mechanism for adenosine delivery, specifically through the generation of short, mucus-penetrating peptides, this study provides a possible theoretical basis for the application of protein–nucleotide complexes in the food and pharmaceutical industries. By overcoming inherent barriers such as poor solubility and low bioavailability, this approach paves the way for developing high-value, plant-based functional foods tailored for specific populations, such as the elderly or those with malabsorption issues. Ultimately, this work contributes to the sustainable utilization of plant resources, aligning with the growing global demand for clean-label and eco-friendly nutraceuticals.

Despite these promising findings, certain limitations of this study should be acknowledged. First, the specific molecular mechanism by which cAMP mediates the absorption of plant-based proteins remains to be fully elucidated. The precise signaling pathways or transporters involved in the cellular uptake of these complexes require deeper investigation. Second, the current evaluation is limited to in vitro cell models. While the Caco-2 monolayer provides valuable insights into intestinal permeability, it cannot fully replicate the complex physiological environment of a living organism, such as dynamic gastrointestinal motility and first-pass metabolism. Therefore, future studies employing in vivo animal models are warranted to validate the bioavailability and transport efficiency of the cAMP-WP complex, thereby bridging the gap between cellular mechanisms and physiological efficacy.

## Figures and Tables

**Table 1:** Concentrations of absorption transport.

Sample	Concentration (mg/mL)		
	30 min	60 min	120 min
ADP-WP	0.013 ± 0.001 ^a^	0.018 ± 0.002 ^a^	0.035 ± 0.011 ^b^
ADP	0.019 ± 0.003 ^a^	0.036 ± 0.007 ^a^	0.088 ± 0.016 ^b^
AMP-WP	0.017 ± 0.006 ^a^	0.026 ± 0.006 ^a^	0.074 ± 0.025 ^b^
AMP	0.004 ± 0.001 ^a^	0.006 ± 0.002 ^ac^	0.013 ± 0.005 ^bc^
ATP-WP	0.006 ± 0.001 ^ac^	0.013 ± 0.004 ^b^	0.010 ± 0.002 ^bc^
ATP	0.025 ± 0.010 ^a^	0.033 ± 0.006 ^a^	0.050 ± 0.022 ^a^
cAMP-WP	1.773 ± 0.402 ^a^	1.357 ± 0.075 ^b^	0.233 ± 0.048 ^b^
cAMP	0.019 ± 0.007 ^a^	0.030 ± 0.002 ^a^	0.047 ± 0.005 ^b^

Different lowercase letters indicate statistical significance (*p* < 0.05).

## Data Availability

The original contributions presented in this study are included in the article/Supplementary Materials. Further inquiries can be directed to the corresponding author.

## Supplementary Materials

The following supporting information can be downloaded at https://www.mdpi.com/article/10.3390/foods15081429/s1.
