# Supplementary material for: Complexation of Walnut Protein with Adenosine Nucleotides: Effects on Protein Functionality and Novel Insight into the Absorption Mechanism of cAMP
Source: Foods. 2026 Apr 20;15(8):1429. doi: 10.3390/foods15081429 (PMC13115319; doi:10.3390/foods15081429)
Supplement: Supplementary file 1 [file foods-15-01429-s001.zip › foods-4200636-supplementary.pdf]

Supplementary Materials

for

Complexation of Walnut Protein with Adenosine Nucleotides: Effects on Protein Functionality and a Novel Insight into the Absorption Mechanism of cAMP

Lei Zhang[1], Shanxing Gao[1], Ye Wang[3], Jingming Li[2], Jiachen Zang[1]*

[1]*College of Food Science and Nutritional Engineering, China Agricultural University, Beijing 100083, China*

[2]*Sichuan Advanced Agricultural & Industrial Institute, China Agricultural University, Chengdu 611430, China*

[3]*Network and Information Technology Center, Xinjiang Agricultural University, Urumqi 830052, China*

**Corresponding author: Jiachen Zang* zangjiachen@cau.edu.cn

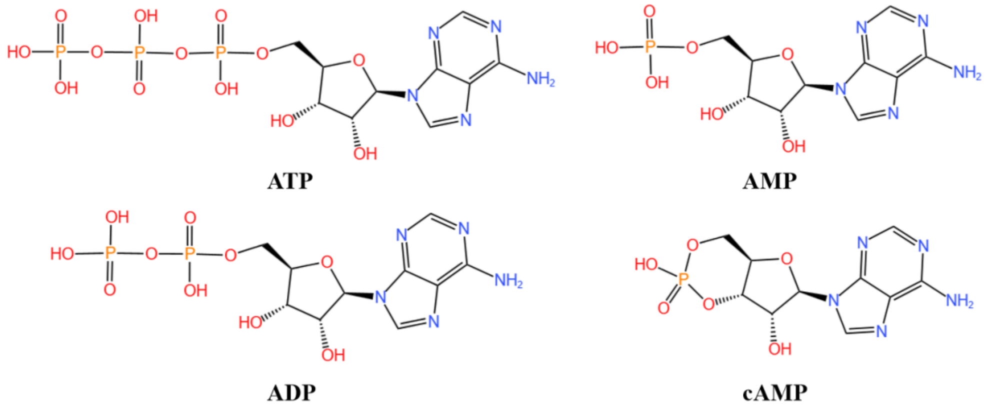

Figure S1. Chemical molecular structures of ATP, ADP, AMP, and cAMP.

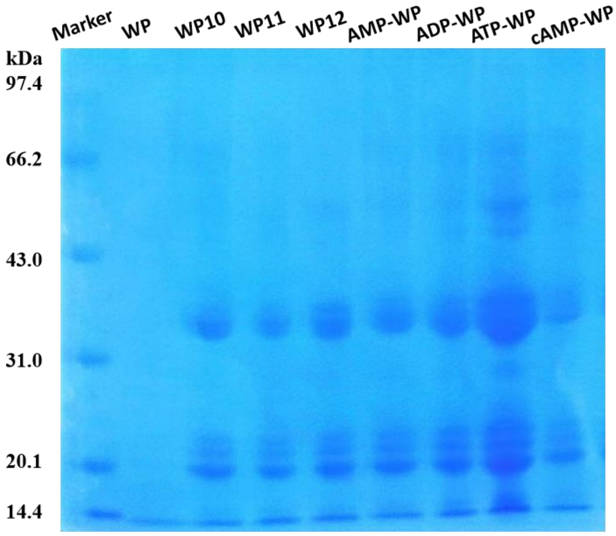

Figure S2. SDS-PAGE of WP, alkaline treated WP and AWP.

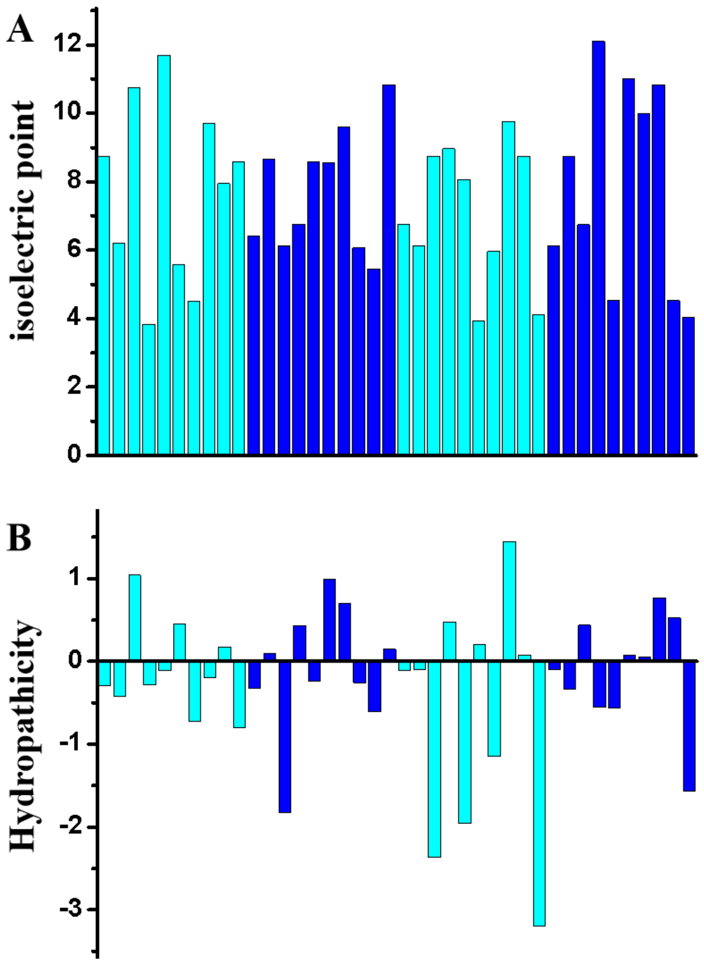

Figure S3. Predicted isoelectric point (A) and average hydropathicity (B) of the peptides derived from digested AWP.

Supplementary Table S1.

Identified peptides obtained from AWP with high scores.

Sample	Identified peptide sequences
ATP-WP	LEMVKLLQPVNNPGQFR<br>MSTAPPFVSASSSPASAPLLDATSHARAHQFHADLDQNPR<br>RMTRNLIIFSVILEVVAVSYAIMTTR<br>DQAILFTLPPLEEETDGMAK<br>ILNELTFSSTRSLLRR<br>MALGDLMASSR<br>VAGTSGKALLEGGSDDEGSSTEAHGR<br>MNKLLLQAPPPYINPHFSHTNSSFIIFDASIQMAKR<br>MQAVIVACDLAQSNLKSVPPKLISQATER<br>KLIPLPPADPLRNYTSGETR
ADP-WP	TVTITPSNQALTEGNAIHK<br>SSCKCTSNCHAWGLVILTAGWDGRIK<br>FVREEQGQR<br>FGAHLVLVLRYLLAEEMKDTFR<br>QGIVLLKNDK<br>VASLGLLPNMILYLMGTYR<br>MKHYTAAISLIKHLFSLGLKPNIYTLSIVVNCLCR<br>GEDAISLNSVAFSQMKR<br>NFYVESPAVRAMSDQEVMHYR<br>HQAVALRIGSDKSVIFR
AMP-WP	LVYYVVQGRGLHGAAIPGCPETFQSESSSQFR<br>FNYLYSLVQVEVETKTAK<br>KQSLREWER<br>IIAQGHYSERAAASLCRSIVNVVHICHFMGVMHR<br>CQRRCQIQEQSPER<br>YIYTIDDDCFVAK<br>MPAMESINQQEHGHNSVK<br>FHILIIATGSTVIR<br>HQAVALRVNADLTAFYR<br>QEEEEEEEARR
cAMP-WP	FNYLYSLVQVEVETKTAK<br>LQKLHDLVR<br>LNLVHPIELK<br>VPGIGHRIKSR<br>VEASTECR<br>KPIGVAGSAR<br>LQVGKKVSDIIR<br>TLVDRLLMAAKSVATIR<br>ILLEEYTSVLR<br>DVCDENTR

Ribosylation ■ Oxidation ■ Carbamidomethylation ■ Acetylation
